# Cold Catching

**Published:** 1882-04

**Authors:** 


### "COLD CATCHING."

IT is noteworthy as a curious, yet easily explicable fact, that few persons take cold who are not either self-consciously careful, or fearful of the consequences of exposure. If the attention be wholly diverted from the existence of danger by some supreme concentration of thought, as, for example, when escaping from a house on fire or plunging into cold water to save life—the effects of "chill" are seldom experienced. This alone should serve to suggest that the influence exerted by cold falls on the nervous system. The immediate effects of a displacement of blood from the surface, and its determination to the internal organs, are not, as was once supposed, sufficient to produce the sort of congestion that issues in inflammation. If it were so, an inflammatory condition would be the common characteristic of our bodily state. When the vascular system is healthy, and that part of the nervous apparatus by which the caliber of the vessels is controlled performs its proper functions normally, any disturbance of equilibrium in the circulatory system which may have been produced by external cold, will be quickly adjusted. It is, therefore, on the state of the nervous system that everything depends, and it is, as we have said, on the nervous system the stress of a "chill" falls. Consciousness is *one* element in the production of a *cold*, and when that is wanting the phenomenon is not very likely to ensue.

It is in this way that persons who do not cultivate the fear of cold catching are not, as a rule, subject to this infliction. This is one reason why the habit of wrapping up tends to create a morbid susceptibility. The mind, by its fear-begetting precaution, keeps the nervous system on the alert for impressions of cold, and the centres are, so to say, panic-stricken when even a slight sensation occurs. Cold applied to the surface, even in the form of a gentle current of air somewhat lower in temperature than the skin, will produce the "feeling" of "chill." Conversely a thought will often give rise to the "feeling" of cold applied to the surface—for example, of "cold water running down the back." Many of the sensations of cold or heat which are experienced by the hypersensitive have no external cause. They are purely ideational in their mode of origination, and ideal in fact.—*Lancet.*

